# Getting the Most Out of Your Crystals: Data Collection at the New High-Flux, Microfocus MX Beamlines at NSLS-II

**DOI:** 10.3390/molecules24030496

**Published:** 2019-01-30

**Authors:** Michelle S. Miller, Sweta Maheshwari, Wuxian Shi, Yuan Gao, Nam Chu, Alexei S. Soares, Philip A. Cole, L. Mario Amzel, Martin R. Fuchs, Jean Jakoncic, Sandra B. Gabelli

**Affiliations:** 1 Department of Oncology, Johns Hopkins University School of Medicine, Baltimore, MD 21205, USA; michelle.miller@jhmi.edu; 2 Department of Biophysics and Biophysical Chemistry, Johns Hopkins University School of Medicine, Baltimore, MD 21205, USA; sweta@jhmi.edu (S.M.); mamzel@jhmi.edu (L.M.A.); 3 Energy & Photon Sciences Directorate, Brookhaven National Laboratory, Upton, NY 11973, USA; wushi@bnl.gov (W.S.); yuangao@bnl.gov (Y.G.); soares@bnl.gov (A.S.S.); mfuchs@bnl.gov (M.R.F.); jjakoncic@bnl.gov (J.J.); 4 Case Center for Synchrotron Biosciences, Case Western Reserve University, Cleveland, OH 44106, USA; 5 Division of Genetics, Departments of Medicine and Biological Chemistry and Molecular Pharmacology, Harvard Medical School and Brigham and Women’s Hospital, Boston, MA 02115, USA; nchu3@bwh.harvard.edu (N.C.); pacole@bwh.harvard.edu (P.A.C.); 6 Department of Medicine, Johns Hopkins University School of Medicine, Baltimore, MD 21205, USA

**Keywords:** multicrystal, vector data collection, NSLS-II, PI3Kα, Akt1, CDP-Chase, PHM, microfocus, microdiffraction, nonhomogeneous crystals

## Abstract

Advances in synchrotron technology are changing the landscape of macromolecular crystallography. The two recently opened beamlines at NSLS-II—AMX and FMX—deliver high-flux microfocus beams that open new possibilities for crystallographic data collection. They are equipped with state-of-the-art experimental stations and automation to allow data collection on previously intractable crystals. Optimized data collection strategies allow users to tailor crystal positioning to optimally distribute the X-ray dose over its volume. Vector data collection allows the user to define a linear trajectory along a well diffracting volume of the crystal and perform rotational data collection while moving along the vector. This is particularly well suited to long, thin crystals. We describe vector data collection of three proteins—Akt1, PI3Kα, and CDP-Chase—to demonstrate its application and utility. For smaller crystals, we describe two methods for multicrystal data collection in a single loop, either manually selecting multiple centers (using H108A-PHM as an example), or “raster-collect”, a more automated approach for a larger number of crystals (using CDP-Chase as an example).

## 1. Introduction

Macromolecular X-ray crystallography has revolutionized the study of proteins, providing atomic level detail that offers insight into fundamental molecular mechanisms and guides drug design. Historically, the rate-limiting step in structure determination has been the growth of crystals of sufficient size and homogeneity to yield high quality X-ray diffraction. Extensive screening and optimization are undertaken, but often fail to yield large, well-diffracting crystals. Now, with the advent of third generation synchrotrons and microfocus beams, the need for such time-consuming optimization is rapidly decreasing [1]. Small (<20 µm) crystals that would have previously been discarded, can now yield diffraction quality data [2]. This dramatically decreases the time required for optimization of crystal conditions and increases productivity of both mechanistic and drug discovery efforts.

The two recently opened beamlines for macromolecular crystallography at NSLS-II have launched the microcrystallography field into new territory, with flux densities up to two orders of magnitude brighter than those previously available [3]. The Frontier Microfocusing Macromolecular Crystallography beamline (FMX, X17-ID-2) has a beam size of 1 × 1.5 µm, expandable to 10 µm and a flux of 3.5 × 10^12^ photons/s. The Highly Automated Macromolecular Crystallography beamline (AMX, X17-ID-1) has a beam size of 5 × 7.5 µm and a flux up to 5 × 10^12^ photons/s [3]. This state-of-the-art technology unlocks new possibilities for microcrystal diffraction and serial crystallography experiments. 

Such small beam sizes make data collection on crystals that are less than 10 µm in size achievable. However, such small crystals can often be optically obscured, particularly in the case of membrane protein crystals grown using lipidic cubic phase (LCP) conditions, and as such it is difficult to optically center these crystals in the X-ray beam [4,5,6]. In addition, the crystals may be thinner than the thickness of the loop and impossible to accurately center by eye when the plane of the sample holder is parallel to the X-ray beam. To address this challenge, automated rastering is available at both AMX and FMX. Rastering is a grid-search technique that takes single, low intensity diffraction images at regular, specified intervals across the loop [5,6,7]. A spot finding algorithm is typically used to generate a heatmap using the diffraction data [8,9,10]. The heatmap is then superimposed onto the image of the loop for easy evaluation. Rastering can be useful not only for the identification of small crystals, but also for finding the best areas to collect from large but nonhomogeneous crystals [6,7].

An associated challenge for microfocus beams is the rapid onset of radiation damage caused by the high intensity beam. To address this challenge, AMX and FMX are equipped with rapid scanning high precision goniometers and provide dedicated collection protocols. Vector collection is a protocol which automatically translates the sample along a user-defined vector with rotation around θ [6,7,11]. The vector is often defined in line with the longest axis of the crystal. This continuously exposes new regions of the crystal to the beam, and is particularly useful for long, thin crystals, and also for smaller well-diffracting volumes of a larger nonhomogeneous crystal. By taking into account an appropriate radiation dose limit [12], vector collection optimally exposes each region of the crystal to the X-ray beam. Both rastering and vector collection have been integrated into the software for automation at FMX and AMX.

For crystals that cannot survive the dose required for the collection of a complete dataset, variations on multicrystal or serial crystallography must be used. Before the advent of cryocooling, serial crystallography was frequently used to overcome total dose damage [13]. More recently, it has been successfully applied to data collected from X-ray Free-Electron Lasers [14]. This combines single images collected from hundreds to thousands of microcrystals each of which is destroyed in the diffraction event. In contrast, serial data collection at synchrotrons allows for rotational data collection and multiple exposures of a single crystal due to the reduced X-ray intensity [15]. Advances in instrumentation at the FMX beamline allow for the integration of raster scanning data collection of multiple microcrystals mounted across the surface of a loop or mesh [16].

In this study, we highlight the capabilities of the AMX and FMX beamlines at NSLS-II by describing structure determination from single crystal vector data collection and two methods for multicrystal data collection. We describe dose management in the context of a sample population that was limited in both size and number, with little spare diffraction potential to waste on either underexposed or overexposed crystal regions. We further describe data processing issues to optimally balance the competing needs for data completeness, data quality, and resolution limit.

## 2. Results

### 2.1. Single Crystal Vector Data Collection

#### 2.1.1. Akt1

**Figure 1:**
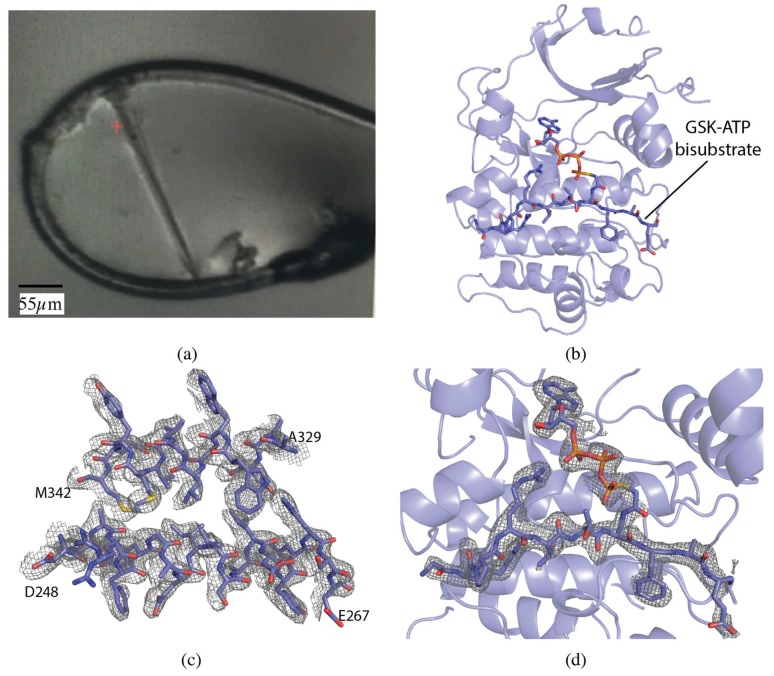
Vector data collection of Akt1 crystals. (**a**) Akt1 crystal in loop. (**b**) Akt1 structure (PDB ID 6NPZ). Ribbon representation of Akt1, with the GSK3-ATP bisubstrate analog shown in stick representation. (**c**) 2mFo-DFc map for a section of Akt1 contoured at 1.0 σ. (**d**) mFo-DFc omit map for GSK3-ATP bisubstrate analog contoured at 3.0 σ.

The serine/threonine protein kinase Akt1 (also known as Protein Kinase B) crystals grew as long, thin rods of approximately 275 × 15 × 15 µm (Figure 1a). The crystal was centered so that the face of the loop was perpendicular to the beam. A vector was defined from one end of the crystal to the other and X-ray diffraction data were collected along the vector in 0.2° slices with a total rotation of 180°. The vector length was 275 µm, at a flux of 8 × 10^11^ photons/s, total exposure time 36 s, with a volume distributed dose of 4 MGy (calculated using RADDOSE-3D [17]). The collected data were indexed, integrated and scaled with FastDP [18,19,20,21]. The resolution cut-off criterion was 〈I/σ(I)〉 of 2.0. The data had an overall R_merge_ of 10.1%, R_pim_ of 4.2%, and CC_1/2_ of 99.6%, with values in the highest resolution shell of 76.0%, 31.4%, and 84.9%, respectively. Completeness was 98.5% to 2.12 Å resolution (Table 1). 

The structure was determined by molecular replacement with MOLREP [22] using PDB 6BUU as the search model [23]. The dataset was refined to a final resolution of 2.1 Å using iterative rounds of refinement with REFMAC5 [21,24] and manual rebuilding in Coot [25] (Figure 1b–d). The overall quality of the model was assessed with Molprobity and wwPDB validation tools.

#### 2.1.2. PI3Kα

**Figure 2:**
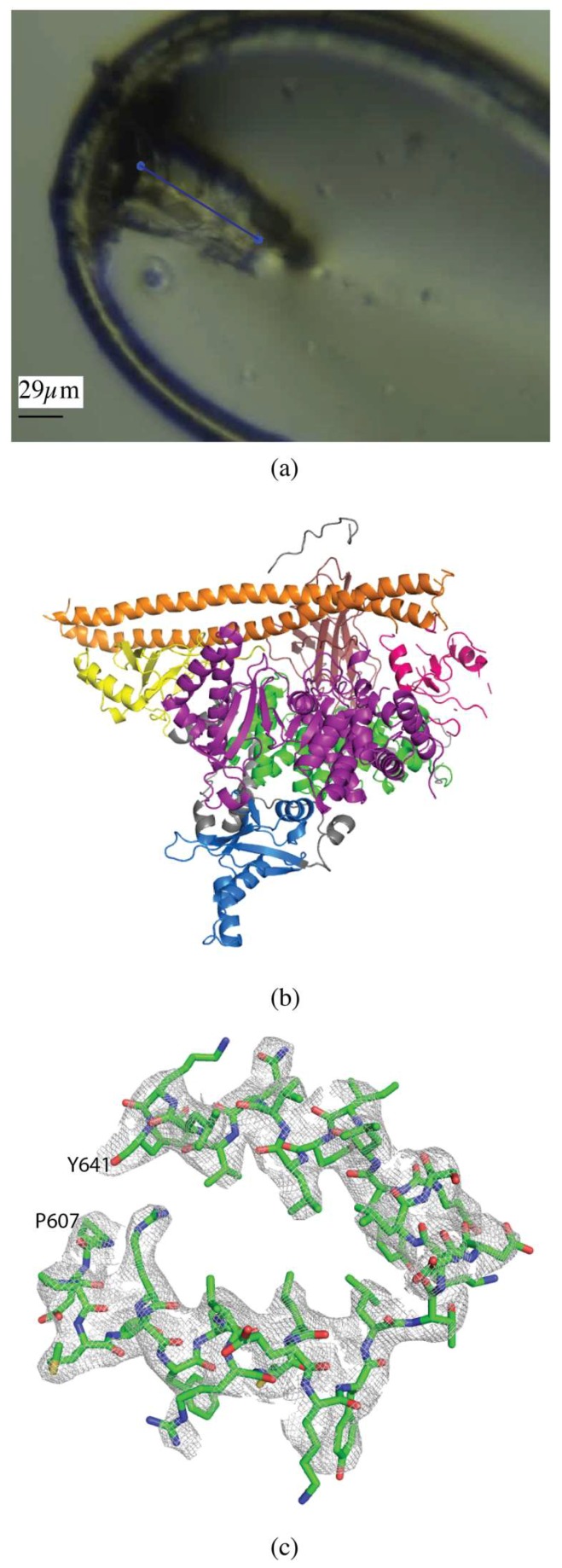
Vector data collection of PI3Kα crystals. (**a**) PI3Kα crystal in loop with the vector for data collection shown as a blue line. (**b**) Overall structure of PI3Kα, colored by domain (PDB ID 6NCT). (**c**) 2mFo-DFc map of a section of the helical domain of the PI3Kα structure contoured at 1.0 σ.

Initial phosphatidylinositol 3-kinase α (PI3Kα) crystals were approximately 5 × 5 × 5 µm, and were grown to an approximate size of 30 × 60 × 15 µm through 5 rounds of macroseeding (Figure 2a). A 60 µm vector was defined along the length of the crystal and data collected in 0.2° slices, over a total rotation of 140°, at a flux of 2 × 10^12^ photons/s, total exposure time 28 s, with a volume distributed dose of 10 MGy (calculated using RADDOSE-3D [17]) (Figure 2a). The data were processed with XDS [19], data were cut-off at 〈I/σ(I)〉 of 1.5 with an overall R_merge_ of 10.4%, R_pim_ of 4.6% and CC_1/2_ of 99.8%, and values in the highest resolution shell of 93.5%, 41.8%, and 68.5%, respectively. Completeness was 96.4% to 3.35 Å resolution (Table 1).

The structure was determined by molecular replacement using MOLREP [22] with PDB 4OVU [26] as the search model. Structure refinement was carried out using iterative rounds of refinement in REFMAC5 [24] with three TLS domains defined (chain A residues -26–1061 and chain B residues 329–435 and 439–597) and manual rebuilding in Coot [25] (Figure 2b,c). Alignment with PDB 4OVU gave an RMSD of 0.68 Å and showed no significant differences, as expected. The overall quality of the model was assessed with Molprobity and wwPDB validation tools.

#### 2.1.3. CDP-Chase

**Figure 3:**
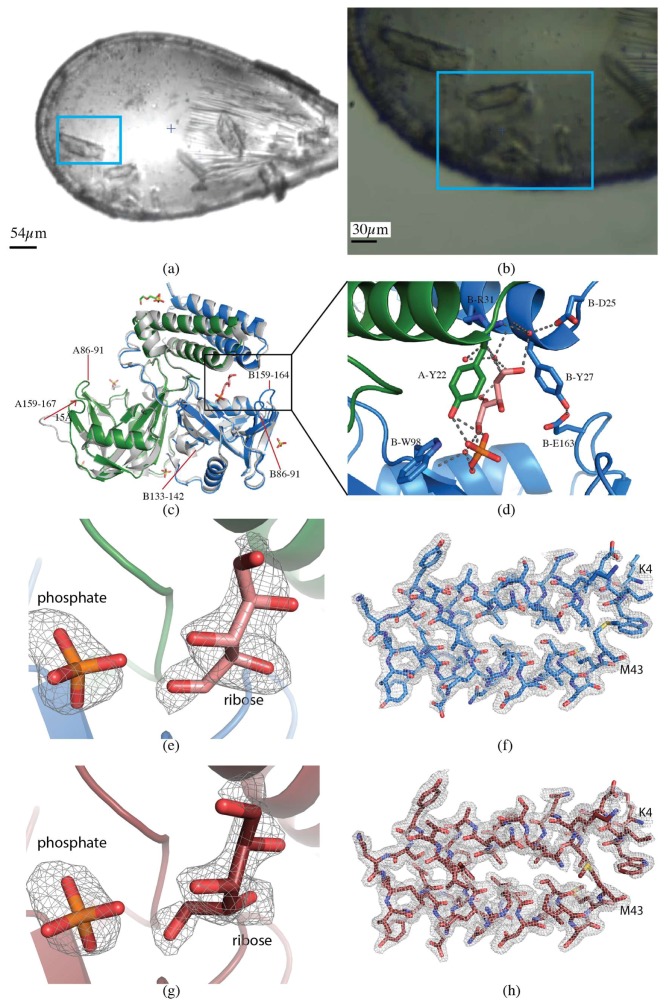
CDP-Chase. (**a**) Single crystal collection. Data were collected from the crystal highlighted in the blue rectangle. (**b**) Multicrystal collection. The raster data collection was performed over the region contained in the blue rectangle. (**c**) Overall structure of CDP-Chase. The alignment of the apo-CDP-Chase (PDB ID 3Q1P), shown in gray, and the structure determined in the presence of ADP-ribose from a single crystal, colored by chain (green and blue, PDB ID 6NCI). The structure determined from multiple crystals is indistinguishable from the single crystal structure, so is not shown. Loops A86-91, B86-91, and B159-164 are ordered in the ADP-ribose structure. Loop B133-142 becomes disordered in the presence of ADP-ribose. Loop A159-167 moves inward by 15 Å in the ADP-ribose structure. (**d**) Zoom in of the ligand binding site. The open ribose molecule is shown in salmon, with hydrogen bonds represented as gray dashed lines. E163, the residue that coordinates the catalytic divalent cation (not in this structure), closes off the binding site by forming a hydrogen bond with Y27. (**e**) mFo-DFc omit map contoured at 3.0 σ for phosphate and ribose molecules in the single crystal dataset (PDB ID 6NCI). (**f**) 2mFo-DFc map contoured at 1.0 σ for a section of the protein in the single crystal dataset (PDB ID 6NCI). (**g**) mFo-DFc omit map contoured at 3.0 σ for phosphate and ribose molecules in the multicrystal dataset (PDB ID 6NCH). (**h**) 2mFo-DFc map contoured at 1.0 σ for a section of the protein in the multicrystal dataset (PDB 6NCH).

Data for the *B. cereus* CDP-Choline Pyrophosphatase (CDP-Chase) crystals were collected on a larger, single crystal (60 × 30 × 20 µm, Figure 3a) using vector collection, and the smaller crystals were used for a multicrystal approach for direct comparison (see Section 2.2.1) (Figure 3b). The vector length was 50 µm, at a flux of 2.5 × 10^11^ photons/s, total exposure time 42 s, with a volume distributed dose of 4.7 MGy (calculated using RADDOSE-3D [17]). For the single crystal, the data were indexed, integrated, and scaled with FastDP to a resolution of 2.08 Å with 〈I/σ(I)〉 of 2.6 [18,19,20,21]. The data had an overall R_merge_ of 13.6%, R_pim_ of 3.9% and CC_1/2_ of 99.8%, with values of 94.2%, 28%, and 81.8% in the highest resolution shell, respectively. Data to 2.08 Å resolution had completeness of 98.8% (Table 1).

The structure was determined by molecular replacement using MOLREP [22] with PDB 3Q1P as the template [27]. The dataset was refined to a final resolution of 2.08 Å using iterative rounds of refinement with REFMAC5 [21,24] and manual rebuilding in Coot [25] (Figure 3c). The overall quality of the model was assessed with Molprobity and wwPDB validation tools.

Extensive efforts to cocrystallize *B. cereus* CDP-Chase in the presence of various substrates have failed. Cocrystals grown in the presence of ADP-ribose, a suboptimal substrate for CDP-Chase, do not show electron density for the complete substrate molecule or the expected product of hydrolysis, phosphoribose. Electron density observed in the active site was consistent with separate ribose and phosphate molecules. The time scale of the crystallization may have allowed for further hydrolysis of phosphoribose by water, forming ribose and phosphate. The ribose was bound directly by B-R31 and formed water-mediated interactions with B-W98 and A-Y22. The phosphate was bound by A-Y22 & B-W98 (Figure 3d). The overall structure aligns well with the published apo structure, with an RMSD of 0.8 Å, but differs in several loops (Figure 3c). When cocrystallized with ADP-ribose, loops A&B 86-91 and B159-164 become ordered, while the loop B133-142 becomes disordered. Perhaps most strikingly, an inward movement of approximately 15 Å was observed in loop A159-167 compared with the corresponding loop in 3Q1P [27] (Figure 3c).

### 2.2. Multicrystal Data Collection

#### 2.2.1. CDP-Chase

The crystals for multicrystal data collection ranged in sizes from 10 × 30 × 10 µm to 30 × 50 × 15 µm (Figure 3b). Diffraction data were collected via the raster scan data collection protocol described previously [16]. The area was scanned five times with starting oscillation angles of 10°, 40°, 70°, 100°, and 130°, with the loop face perpendicular to the X-ray beam at the starting angle of 70°. The flux was 2.5 × 10^11^ photons/s, and a cumulative dose of 3.0 MGy (calculated using RADDOSE-3D [17]). A total of 119 partial datasets were collected and processed using the Python scripted workflow described previously [16] relying on DOZOR [9], XDS [19], POINTLESS, and AIMLESS [21]. The resolution cut-off criterion was 〈I/σ(I)〉 of 1.8. The overall R_merge_ of the final merged dataset was 35.7%, R_pim_ was 13.7%, and CC_1/2_ was 94.1%, with 111%, 42.5%, and 60.8% in the highest resolution shells, respectively. Completeness was 96.6% to 2.0 Å resolution (Table 1). 

The structure was determined by molecular replacement using MOLREP [22] with PDB 3Q1P as the template [27]. The dataset was refined to a final resolution of 2.0 Å using iterative rounds of refinement with REFMAC5 [21,24] and manual rebuilding in Coot [25]. The single and multicrystal structures aligned with an RMSD of 0.11 Å and have a comparable level of detail in the electron density maps (Figure 3c,e–h). The overall quality of the model was assessed with Molprobity and wwPDB validation tools.

#### 2.2.2. H108A-PHM

**Figure 4:**
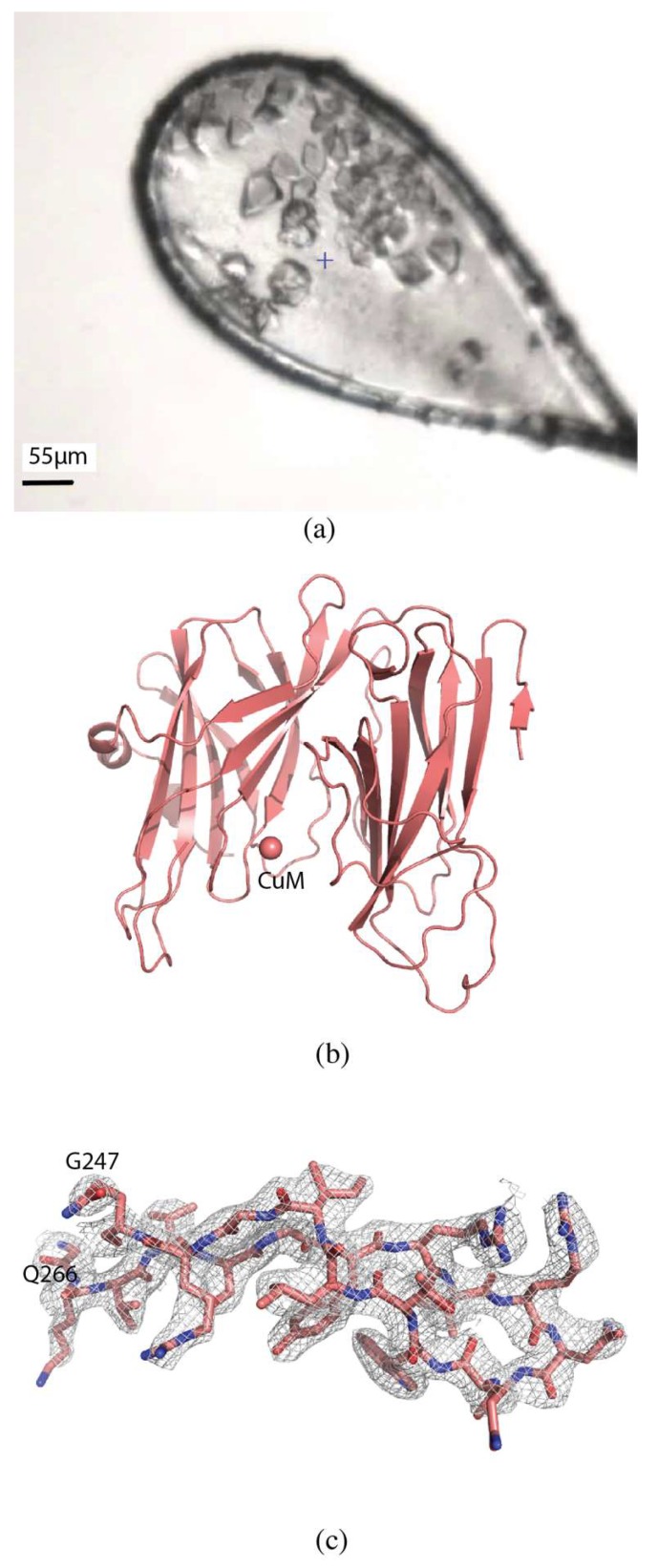
Serial data collection of H108A-PHM. (**a**) H108A-PHM crystals in loop. (**b**) Overall structure of H108A-PHM (PDB ID 6NCK). The single copper atom is shown as a sphere at the Cu_M_ binding site. (**c**) 2mFo-DFc map contoured at 1.0 σ of a section of the protein.

Crystals of H108A-peptidylglycine α-hydroxylating monooxygenase (PHM) were approximately 15 × 25 × 10 µm in size (Figure 4a). Diffraction data were collected at the NSLS-II beamline AMX (17-ID-1). The loop was centered so that the face was perpendicular to the direction of the beam. Thirteen crystals were identified in the loop and multiple centers defined for collection of partial datasets with 0.2° oscillation and a total rotation of 20° to avoid overlapping of crystals. The flux was 4 × 10^12^ photons/s, total exposure time 28 s, with a volume distributed dose of 12 MGy (calculated using RADDOSE-3D [17]). The datasets were processed individually using XDS [19] and seven datasets were selected and scaled with XSCALE to give a final dataset to 2.7 Å resolution with 〈I/σ(I)〉 of 2.8, with overall R_merge_ of 13.0%, R_pim_ of 6.2%, and CC_1/2_ of 99.1%, with values of 58.0%, 30.9%, and 83.0% in the highest resolution shell, respectively. Completeness was 99.6% to a resolution of 2.7 Å (Table 1). 

The structure was determined by molecular replacement using MOLREP [22] with PDB 6AO6 as the search model. The structure was refined using iterative rounds of refinement with REFMAC5 [24] and manual rebuilding using Coot [25] (Figure 4b,c). Alignment with PDB 6AO6 gives an RMSD of 0.60 Å, and shows no significant differences, as expected. The overall quality of the model was assessed with Molprobity and wwPDB validation tools.

## 3. Discussion

### 3.1. Biological Insights 

The PI3K signaling pathway—which regulates cell growth, metabolism, and cell cycle progression—has been the focus of intense interest due to its frequent dysregulation in cancer, diabetes and autoimmune disorders [28]. PI3Kα, one of the four classic Class I isoforms, is activated by growth factor binding to receptor tyrosine kinases, like the insulin receptor, producing the phospholipid, phosphatidylinositol-3,4,5-trisphosphate (PIP_3_). PIP_3_ recruits the protein kinase Akt1 to the plasma membrane, which is fully activated upon phosphorylation of its activation loop by PDK1 and C-terminal tail by mTORC2 [29]. Herein we describe the determination of the structure of two key members of this pathway, PI3Kα and Akt1. We have previously published a series of PI3Kα structures, including the holoenzyme, the complex with a lipid substrate analog, and soaked crystal complexes from a fragment-based drug design effort [26,30,31]. These structures were determined from crystals grown to an average length of >100 µm, which required an average of 6–8 rounds of macroseeding. Due to advances in synchrotron technology (discussed in more detail below) this present structure could be determined from a crystal of 60 µm, thus reducing the rounds of macroseeding required to obtain diffraction quality crystals. We have described the structure of Akt1 bound to a bisubstrate analog elsewhere [23], which revealed vital insights into the role of C-terminal phosphorylation in the activation mechanism of the protein kinase Akt1. 

PHM is a dicopper enzyme involved in peptide amidation: it hydroxylates the C-terminal glycine Cα in the first step of a sequential two-step process to produce the amidated peptide product [32]. Amidated peptides are frequently secreted and function as hormones, neurotransmitters, and growth factors [33]. Our lab has previously published a series of wild type and mutant PHM structures that investigate the role of copper occupancy on catalysis and substrate binding [33]. The crystal conditions for H108A-PHM produced crystals of two sizes; the larger crystals were used to collect the single crystal dataset published previously (PDB ID 6AO6) [33]. This present structure was determined from a series of seven smaller crystals, averaging approximately 25 µm in size, collecting 20° of data from each crystal. The structures align well, with an RMSD of only 0.6 Å.

*B. cereus* CDP-Chase is a member of the Nudix hydrolase superfamily, which are a family of enzymes that hydrolyze **nu**cleoside **di**phosphates linked to some other moiety, **x** [34,35]. It is a bifunctional enzyme that can hydrolyze CDP-choline with the typical nudix reaction in addition to a non-nudix exonuclease reaction on RNA [27]. Despite extensive efforts, substrate-bound structures of CDP-Chase have remained elusive. Although its endogenous substrate is CDP-choline, CDP-Chase can also hydrolyze other nudix substrates including ADP-ribose. Here, we have described the determination of two CDP-Chase structures cocrystallized with ADP-ribose. Some key differences in several loops were observed. Interestingly, the biggest change was in loop A159-167, which bears the glutamate, E163, that completes the coordination of the catalytic divalent cation. The distance between the Cα atoms of E163 in the apo- and ADP-ribose structures is 15 Å (Figure 3d). It appears as if the loop has moved inward to bind the divalent cation and substrate, remaining in the “active” conformation post-hydrolysis. The apo and ADP-ribose CDP-Chase crystals have the same space group and similar cell dimensions, discarding the possibility that the loop movement is caused by differing crystal contacts. Overall, the ordering of previously disordered loops and the sizable changes in the conformations of other key catalytic loops suggests that these crystals have captured another catalytically relevant snapshot of CDP-Chase, most likely the active conformation.

### 3.2. Data Collection Strategies 

The advent of next generation synchrotron beamlines [36] has necessitated the development of new strategies to fully benefit from the increased beam intensity and smaller beam sizes while minimizing radiation damage [2]. The different data collection strategies highlighted in this work demonstrate the variety of options MX beamline users can choose to collect data from their crystals. In the following, we give a guideline for picking an optimal strategy for the sample at hand.

A multitude of factors contribute to a successful data collection. These begin with aspects involved in crystal mounting: which sample pin is used, how well the buffer around the crystal is removed, and the choice of the proper cryoprotection conditions. Ideally, the user will optimize these variables before visiting the synchrotron.

The crystallographic status of each project is also a deciding factor in choosing the collection strategy. Is this a first characterization of new crystals, is the aim to increase the resolution of an existing structure, or is it a study to investigate ligand binding in a known structure? Does a homology model for molecular replacement exist, or is this a de novo phasing project? For example, for first characterizations and for increasing the resolution, a researcher may choose to accept lower data completeness from a crystal, while anomalous collections can adopt moderate resolution but benefit from higher multiplicity. Optimal strategies for these and other objectives have been discussed previously [37,38,39], all with the aim to obtain the best data from the allowable dose before the onset of excessive radiation damage [12,40]. 

Most beamlines let users choose flux, size, and energy. While the choice of energy is guided by resolution and anomalous scattering criteria, crystal size, shape, and morphology should be considered to determine the beam size. Essentially, the beam size should match the best diffracting homogeneous volume of the crystal [41,42]. This can be determined by an initial microbeam raster collection at a low beam intensity to provide detailed maps of the diffraction quality of crystals [4,5,6]. Above a beam size of 10–20 µm, however, a point of diminishing returns is reached for all but the largest unit cell size crystals.

The crystal size and buffer composition, as well as the space group and unit cell size if known from earlier collections, are needed to calculate a good dose rate estimate. The overall dose delivered by a beam with a given profile, energy and flux, along with the dose distribution over a crystal of a given size can be modeled by the program RADDOSE-3D [17]. In microfocus crystallography, one often works with beams smaller than the overall crystal size. In that case, it becomes important to consider the dose distribution over the volume of a crystal in addition to the overall dose. The dose delivered should stay well below the damage limit for specific radiation damage, which can vary greatly depending on the sequence, chemical composition of the buffer, and solvent content [43]. Ideally, a sacrificial crystal, or the corner of a larger one, can be spent on determining the radiation sensitivity empirically. To set the dose rate for an experiment, one can vary exposure time or beam attenuation. Provided the goniometer and sample holder support the required rotation and scanning speeds, and the detector can handle the photon counts and the frame rate, we find that there is no downside to increasing the flux and lowering the exposure times, to benefit from the shorter data collection times [16].

Crystal morphology also influences the choice of the best data collection strategy. In the case of needles the best strategy is a vector data collection. In vector collection, also known as helical data collection [6,11], a size-matched beam is translated along the needle, to evenly distribute the allowable X-ray dose over the volume of the crystal. The collection of data on the Akt1 crystal is a perfect example of this (Figure 1a). If a stationary data collection were attempted for the same crystal, a compromise in beam size would have to be made. This would expose buffer solution to the beam, increasing background scattering and reducing the achievable resolution of the sample. For samples with unknown radiation sensitivity, one can execute a stepwise exposure along the needle, and later choose the undamaged frames to merge for a complete dataset.

Several options should be considered for plate shaped crystals. For example, several parallel vector trajectories can be defined to cover the plate. In this case, the crystal orientation with the beam through the length of the plate is impossible to collect without overlapping with the parallel vectors, so it should be excluded, or collected from an edge that is spared by the vectors. An alternative method is to vary the beam size to match it to the rotation-dependent projection of the plate [44]. A protocol for a rotation-dependent beam size change by using slits is planned at AMX and FMX but has not yet been commissioned. If an average beam size is selected, this has the disadvantage of illuminating the surrounding buffer and adding unwanted background scatter.

For other crystal morphologies, vector collection will often still yield the best results. For example, for the PI3Kα crystal, vector collection along a 60 µm vector uses the full volume of the crystal to allow for the collection of better data (Figure 2a).

For crystals too small to yield a complete undamaged dataset, data must be collected from multiple crystals and the partial datasets merged. In both of the alternatives we present here, multiple crystals can be loaded in a typical sample loop, without requiring the purchase of specialized loops. In the case of H108A-PHM, a multicentering approach was used to collect the data and then the individual datasets were processed and scaled to give a complete dataset. This option works well for cases with a handful of small, easily distinguishable crystals that will survive 10 to 20° of data collection. If enough small crystals are available to achieve high redundancy in serial crystallography, cluster analysis algorithms can be used to select for the optimal set of subdatasets [45,46,47].

For the sake of comparison between the single and multicrystal data collection methods, we collected data of CDP-Chase on both a single crystal and using the raster data collection. As might be expected, the R_merge_ is much higher in the multicrystal dataset compared with the single crystal (35.7% compared with 13.6%). Since R_merge_ fails to take into account higher redundancy [48], both the R_pim_ [48] and CC_1/2_ [49] were considered and found to be reasonable (13.7% and 94.1%, respectively), emphasizing the need to look at multiple measures to assess data quality, particularly in the case of datasets derived from multiple crystals. The single crystal dataset was refined to 2.1 Å (R/R_free_: 0.171/0.222), and the multicrystal dataset to 2.0 Å (R/R_free_: 0.200/0.261) (Table 1). The level of detail observed in both electron density maps is comparable, and the RMSD between the refined structures is 0.11 Å (Figure 3c,e–h). A ribose and a phosphate molecule are clearly defined in both electron density maps (Figure 3f,h). Despite the less favorable statistics for the multicrystal dataset in both the collection and refinement stages, the similarities of the two structures highlight the reliability and applicability of this method when larger crystals are not available, even for ligand or small molecule bound structures.

Radiation damage can be tested in a variety of ways. For example, for the single crystal CDP-Chase dataset, which received a volume distributed dose of 4.7 MGy, well below the Garman limit of 30 MGy [12,40], we evaluated the data for radiation damage. The dataset was divided into four sections and each processed individually. The cell dimensions and mosaicity were stable throughout the entire dataset. We compared the decarboxylation of glutamates and aspartates between the single and multicrystal datasets, and no significant differences were observed. As a result, we concluded that this crystal does not display significant signs of specific radiation damage.

The continuing development of brighter synchrotron sources holds significant promise for structure determination. In conjunction with brighter sources, the improvement of data collection strategies opens up new possibilities for structure determination from previously intractable crystals. The high flux and small beam size of the new beamlines allow the possibility to raster scan all crystals to obtain high-resolution information about diffraction quality over their complete volume. The high dose rates, in conjunction with the newly developed ultrafast raster scanning goniometer [16] brings formerly time-consuming serial crystallography measurements into the minutes range. These new opportunities mean that inhomogeneous large crystals can now deliver high quality data collected from their best volume, and crystals as small as 5 to 10 µm can deliver one or more complete datasets [2,50].

## 4. Materials and Methods

### 4.1. Protein Expression, Purification, and Crystallization

Details for the expression, purification, and crystallization of Akt1 have been reported elsewhere [23]. Briefly, Akt1 (aa.123–459) was produced as the fusion construct, Akt1-*Mxeintein*-CBD, in Sf9 cells. The cells were lysed, the clarified supernatant added to a bed of fibrous cellulose, filtered, loaded onto a chitin resin, and eluted with MESNA. The recombinant thioester fragment was phosphorylated on Thr308 by GST-PDK1 and then the synthetic, N-Cys containing C-terminal phosphorylated peptide was ligated to the cleaved Akt1 thioester. Semisynthetic Akt1 (aa.123–480) with pThr308 and C-terminal triple-phosphorylated residues Ser473, Ser477, and Thr479 (10 mg/mL) was mixed with 1 mM GSK3-ATP bisubstrate and 1 mM MnCl_2_ and crystallized using sitting-drop vapor diffusion at 4 °C with 0.1 M Hepes pH 7.5, 12.5% PEG3350, 0.2 M ammonium acetate. Crystals were cryoprotected with 20% (*v/v*) of 50% PEG3350 added to the reservoir solution, cryocooled and stored in liquid nitrogen until data collection.

PI3Kα (p110α/niSH2) was expressed in Sf9 cells, purified and crystallized as previously reported [30,51]. Briefly, Sf9 cells were co-infected with viruses for N-term 6xHis-p110α and niSH2-p85α, and the cell pellet harvested after 48 h. The complex was purified via stepwise immobilized metal affinity chromatography, anion exchange, and size-exclusion chromatography. Crystals were obtained via hanging drop vapor diffusion using a reservoir containing 0.1 M Hepes pH 7.0, 1.50–1.75 M sodium formate. Multiple rounds of macroseeding were required for diffraction quality crystals.

*B. cereus* CDP-Chase was expressed in *E. coli* and purified as previously described [27]. CDP-Chase (10 mg/mL) was mixed with 1 mM ADP-ribose and cocrystals were grown by hanging drop vapor diffusion with the reservoir containing 0.1 M Tris-HCl pH 8.5, 25–30% PEG 4000, and 0.2 M Li_2_SO_4_. Crystals were cryocooled without the addition of cryoprotectant.

H108A-PHM was expressed, purified, and crystallized as described previously [33]. Briefly, the protein was secreted from stably transfected CHO cells. Crystals were obtained with purified protein by hanging drop vapor diffusion with a reservoir containing 0.1 M Tris-HCl pH 8.5, 0.54 M MgCl_2_, and 19–24% PEG 4000.

### 4.2. Data Collection

Data for Akt1, PI3Kα, and H108A-PHM were collected at the National Synchrotron Light Source II (Upton, NY, USA) beamline 17-ID-1 on a DECTRIS Eiger 9M Detector. Data for CDP-Chase was collected at National Synchrotron Light Source II (Upton, NY, USA) beamline 17-ID-2 on a DECTRIS Eiger 16M Detector.

### 4.3. Accession Codes

The PDBs have been deposited with the following accession codes; PI3Kα 6NCT, CDP-Chase (single crystal) 6NCI, CDP-Chase (multicrystal) 6NCH, and H108A-PHM 6NCK.

## Figures and Tables

**Table 1:** Data collection and refinement statistics.

	Akt1 6NPZ	PI3Kα 6NCT	CDP-Chase/ADP-Ribose (Single) 6NCI	CDP-Chase/ADP-Ribose (Multi) 6NCH	H108A-PHM 6NCK
**Data Collection**					
Diffraction source	NSLS-II X17-ID-1	NSLS-II X17-ID-1	NSLS-II X17-ID-2	NSLS-II X17-ID-2	NSLS-II X17-ID-1
Wavelength (Å)	0.99962	0.919909	0.97934	0.97934	0.918394
Beam size (µm)	8 × 6	7 × 5	5 × 6	5 × 6	7 × 5
Temperature (K)	100	100	100	100	100
Detector	Dectris Eiger 9M	Dectris Eiger 9M	Dectris Eiger 16M	Dectris Eiger 16M	Dectris Eiger 9M
Rotation range per image (°)	0.2	0.2	0.2	0.2	0.2
Total rotation range (°)	180	140	280	119	140
Space group	p2_1_	p2_1_2_1_2_1_	p2_1_2_1_2_1_	p2_1_2_1_2_1_	p2_1_2_1_2_1_
*a*, *b*, *c *(Å)	86.32, 56.09, 92.02	115.36, 117.72, 151.33	61.77, 67.01, 111.43	61.71, 67.20, 111.29	59.31, 65.88, 69.75
α, β, γ (°)	90.00, 104.56, 90.00	90.00, 90.00, 90.00	90.00, 90.00, 90.00	90.00, 90.00, 90.00	90.00, 90.00, 90.00
Resolution range (Å)	29.82–2.12 (2.17–2.12)	49.54–3.35 (3.47–3.35)	29.47–2.08 (2.13–2.08)	19.76–2.00 (2.05–2.00)	47.89–2.70 (2.80–2.70)
Total no. of observations	326,363	159,060	369,062	213,498	38,364
No. of unique observations	48,316	29,228	28,297	30,674	7872
Completeness (%)	98.5 (81.8)	96.4 (97.2)	98.8 (84.5)	96.6 (96.8)	99.6 (99.3)
Redundancy	6.8 (6.6)	5.4 (5.4)	13.0 (11.2)	7.0 (7.0)	4.9 (4.2)
〈*I*/σ(*I*)〉	11.3 (2.0)	11.8 (1.5)	14.4 (2.6)	4.3 (1.8)	8.2 (2.8)
R_merge_	0.101 (0.760)	0.104 (0.935)	0.136 (0.942)	0.357 (1.11)	0.130 (0.580)
R_p.i.m._	0.042 (0.314)	0.046 (0.418)	0.039 (0.280)	0.137 (0.425)	0.062 (0.309)
CC_1/2_	0.996 (0.849)	0.998 (0.685)	0.998 (0.818)	0.941 (0.608)	0.991 (0.830)
**Refinement**					
Resolution range (Å)	89.07–2.12 (2.17–2.12)	49.54–3.35 (3.44–3.35)	29.47–2.08 (2.13–2.08)	19.76–2.00 (2.05–2.00)	44.08–2.70 (2.77–2.70)
No. of reflections, working set	45,932	27,765	26,741	29,211	7477
No. of reflections, test set	2364	1462	1502	1461	394
R_work_/R_free_	0.185/0.241 (0.249/0.316)	0.199/0.270 (0.314/0.343)	0.171/0.222 (0.241/0.291)	0.200/0.261 (0.276/0.334)	0.219/0.294 (0.322/0.342)
**No. of non-H atoms**					
Protein	5406	10,462	3288	3311	2350
Ligand/ion	178	31	52	25	2
Water	475	0	255	366	20
**R.m.s. deviations**					
Bonds (Å)	0.016	0.013	0.009	0.008	0.008
Angles (°)	2.06	1.95	1.51	1.45	1.54
**Average B factors (Å^2^)**					
Protein	41.7	133.8	35.7	27.6	58.9
Ligand/ion	56.2	189.2	78.0	51.8	109.4
Water	45.8	n/a	40.9	34.7	41.6
**Ramachandran (%)**					
Favorable	96.4	95.1	97.2	96.9	95.6
Allowed	2.0	4.5	2.6	3.1	4.4
Disallowed	1.6	0.4	0.2	0	0
